# Persistent Hiccups as an Atypical Sole Manifestation of ST‐Segment Elevation Myocardial Infarction: An Interesting Case Report

**DOI:** 10.1155/cric/1123999

**Published:** 2026-02-20

**Authors:** Osman Farah Dahir, Said Abdirahman Ahmed, Ahmed Elmi Abdi, Ahmed Shafie Aden, Ishak Ahmed Abdi, Mohamud Mire Waberi, Mohamed Omar Hassan, Mohamed Abdullahi Mohamud, Abdullahi Mohamed Hassan Fujeyra, Mohammed A. M. Ahmed, Salad Mahamud Mahamed, Mohamed Sheikh Hassan, Feyza AKSU, Abdijalil Abdullahi Ali

**Affiliations:** ^1^ Department of Cardiology, Mogadishu Somali-Turkish Training and Research Hospital, Mogadishu, Somalia; ^2^ Department of Cardiology, Jazeera Specialist Hospital, Mogadishu, Somalia; ^3^ Faculty of Medicine and Surgery, Somali National University, Mogadishu, Somalia, snu.edu.so; ^4^ Faculty of medicine, Mogadishu University, Mogadishu, Somalia and Department of Pediatric Cardiology, Mogadishu Heart Center, Mogadishu, Somalia; ^5^ Department of Emergency, Mogadishu Somali-Turkish Training and Research Hospital, Mogadishu, Somalia; ^6^ Department of Neurology, Mogadishu Somali-Turkish Training and Research Hospital, Mogadishu, Somalia; ^7^ Department of Cardiovascular Surgery, Mogadishu Somali-Turkish Training and Research, Mogadishu, Somalia

**Keywords:** atypical presentation, inferior wall MI, percutaneous coronary intervention, persistent hiccups, right coronary artery occlusion, ST-elevation myocardial infarction

## Abstract

**Background:**

Myocardial infarction (MI) commonly presents with chest pain, dyspnea, diaphoresis, and nausea; however, atypical and nonclassical presentations are increasingly recognized and may delay diagnosis and management. Persistent hiccups as the sole presenting manifestation of ST‐elevation myocardial infarction (STEMI) are exceedingly rare, with only a few cases reported. Awareness of such unusual presentations is essential to avoid missed or delayed diagnoses.

**Case Presentation:**

A 60‐year‐old male presented to the outpatient clinic with a 1‐month history of persistent hiccups without chest pain or other associated symptoms. He had no significant past medical history, cardiovascular risk factors, or prior medication use. Physical examination and vital signs were within normal limits. Laboratory evaluation revealed markedly elevated cardiac troponin levels (20 ng/mL), whereas creatine kinase, AST, lactate dehydrogenase, and B‐type natriuretic peptide levels were within normal ranges. Electrocardiography showed normal sinus rhythm with ST‐segment elevation and deep *Q* waves in the inferior leads, more prominent in Lead III than Lead II, suggestive of right coronary artery (RCA) involvement. Transthoracic echocardiography demonstrated preserved left ventricular systolic function (ejection fraction 64%) with inferior wall hypokinesia. Urgent coronary angiography revealed total occlusion of the distal RCA, with no significant disease in the left anterior descending or circumflex arteries. The patient underwent successful percutaneous coronary intervention with balloon angioplasty and drug‐eluting stent implantation, resulting in restoration of coronary blood flow. His hospital course was uneventful, and he was discharged on dual antiplatelet therapy planned for 12 months.

**Discussion:**

The proposed mechanism of hiccups in inferior wall MI involves irritation of the phrenic or vagus nerves due to ischemia or inflammation near the diaphragm, particularly in RCA territory infarctions. Review of previously reported cases demonstrates a consistent association between persistent hiccups and inferior wall ischemia, most often related to RCA occlusion. This case reinforces the need to consider cardiac etiologies in patients with unexplained or refractory hiccups, even in the absence of classical ischemic symptoms.

**Conclusion:**

Persistent hiccups may represent a rare but important atypical presentation of inferior wall STEMI. Early cardiac evaluation, including electrocardiography and cardiac biomarkers, should be considered in such cases to enable timely diagnosis and intervention, potentially improving patient outcomes.

## 1. Introduction

Acute coronary syndrome (ACS) encompasses a spectrum of clinical conditions resulting from acute myocardial ischemia, most commonly due to coronary artery obstruction. This spectrum includes unstable angina, non–ST‐segment elevation myocardial infarction (NSTEMI), and ST‐segment elevation myocardial infarction (STEMI). Among these, STEMI represents a time‐critical cardiovascular emergency, classically presenting with acute chest pain, dyspnea, diaphoresis, and nausea [1, 2].

Importantly, a substantial proportion of patients do not exhibit typical ischemic symptoms. Atypical presentations—such as abdominal discomfort, unexplained fatigue, syncope, or silent myocardial infarction (MI)—are well documented, particularly among older adults, individuals with diabetes, and women. Exceptionally rare manifestations may further obscure diagnosis; persistent hiccups represent one of the most unusual and easily overlooked symptoms of STEMI, posing a significant diagnostic challenge [3].

Hiccups, or singultus, are caused by involuntary contractions of the diaphragm followed by sudden closure of the glottis. While they are generally benign and self‐limiting, persistent hiccups lasting longer than 48 h warrant investigation for underlying pathology. These may include central nervous system disorders, metabolic disturbances, gastrointestinal conditions, and, in rare cases, cardiovascular events. Inferior wall MIs are particularly relevant because of their anatomical proximity to the diaphragm and the involvement of the vagus or phrenic nerve, which can trigger hiccups [4].

Several recently reported cases and clinical reviews have suggested that persistent hiccups may, in very rare instances, be the only presenting symptom of STEMI, leading to significant delays in diagnosis and treatment. Early recognition of such an atypical manifestation is vital to initiate timely reperfusion therapy and prevent adverse outcomes. In this case report, we present a patient whose only symptom of an acute ST‐elevation MI was persistent hiccups, emphasizing the need for high clinical suspicion even in the absence of chest pain [5].

The pathophysiological mechanism is thought to be related to irritation of the vagus or phrenic nerve by ischemic myocardial tissue or by associated inflammatory mediators. This nerve irritation can lead to the diaphragm′s involuntary contractions, manifesting clinically as hiccups. Awareness of this potential connection is important in preventing misdiagnosis and improving patient outcomes [6].

Recognition of atypical STEMI presentations such as persistent hiccups is especially crucial in resource‐limited settings where advanced diagnostic tools may not be immediately available. Clinicians must rely on thorough clinical evaluation and maintain a broad differential diagnosis when encountering unexplained persistent hiccups [7].

## 2. Case Presentation

A 60‐year‐old male presented to the outpatient clinic with a 1‐month history of persistent hiccups. He reported no other associated symptoms. He had no history of chronic medical illness, no prior medication use, and no history of smoking, alcohol consumption, or other harmful habits. His family history was unremarkable for chronic disease.

On physical examination, all vital signs were within normal limits. Cardiovascular examination was normal, and examination of other systems revealed no abnormalities.

**Figure 1:**
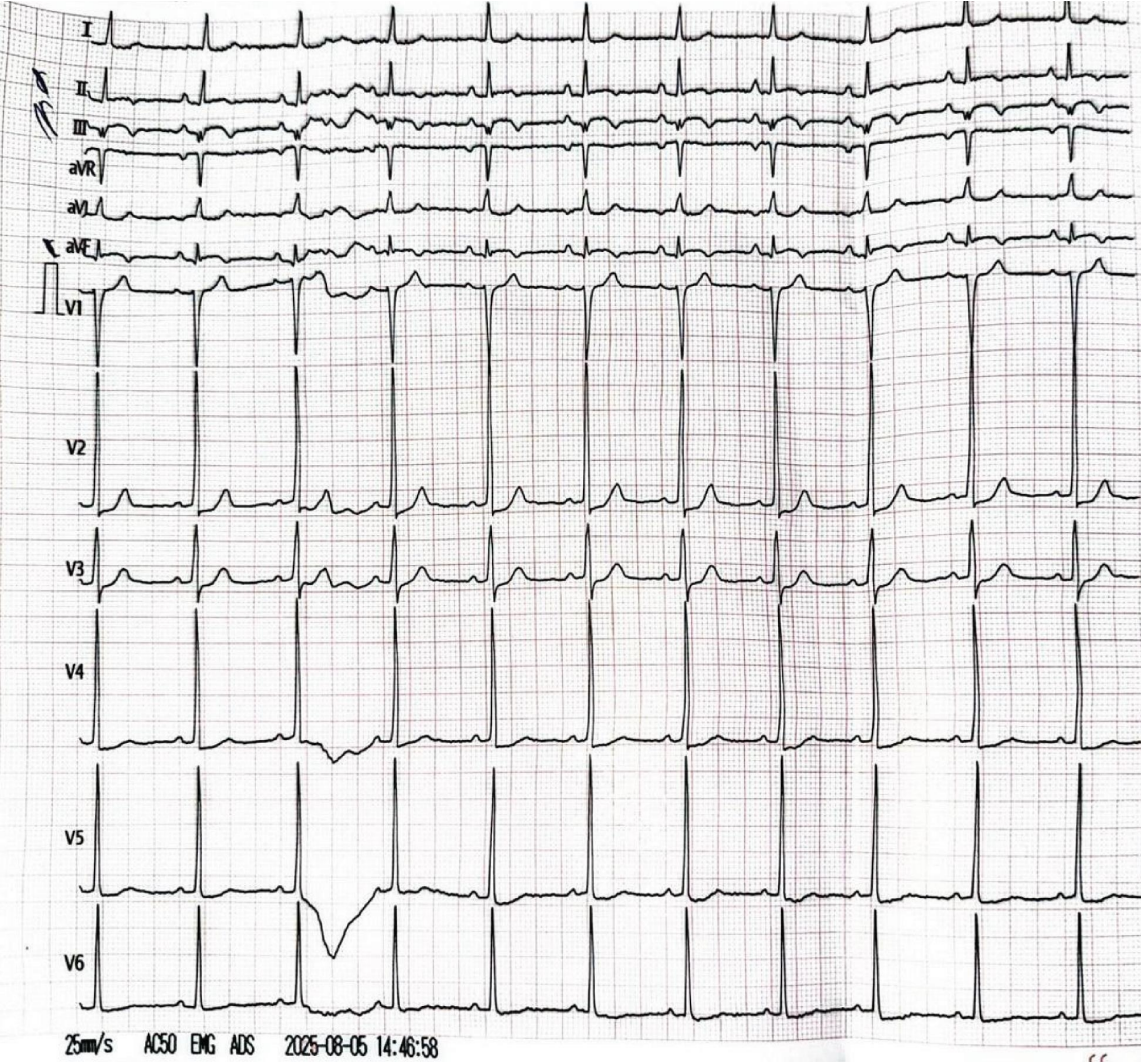
The electrocardiogram (ECG) showed normal sinus rhythm with ST elevation and Q waves in the inferior leads.

Initial laboratory investigations revealed a markedly elevated troponin level of 20 ng/mL (reference range: 0.010–0.029 ng/mL), CK of 44 U/L, AST of 33 U/L, and LDH of 143 U/L. The B‐type natriuretic peptide (BNP) level was 30 pg/mL (normal < 100 pg/mL). Additional laboratory tests, including complete blood count (CBC) and thyroid‐stimulating hormone (TSH), were within normal limits. A chest X‐ray (CXR) showed no acute abnormalities. Electrocardiography (ECG) demonstrated normal sinus rhythm with ST‐segment elevation and deep *Q* waves in the inferior leads, with greater ST‐segment elevation in Lead III than in Lead II, suggesting right coronary artery (RCA) involvement (Figure 1). Transthoracic echocardiography revealed preserved left ventricular systolic function with an ejection fraction of 64%, along with hypokinesia of the inferior wall. No other abnormalities were noted.

Following these findings, the patient was administered loading doses of aspirin (300 mg) and clopidogrel (600 mg). He was then referred for urgent cardiac catheterization. Coronary angiography revealed a total occlusion of the distal RCA (Figure 2).

Figure 2The left anterior oblique (LAO) caudal angiographic view demonstrates a 100% occlusion of the distal right coronary artery (RCA). (a) The left anterior oblique (LAO) caudal angiographic view shows balloon dilation performed in the distal right coronary artery (RCA). (b) Following balloon dilation, angiographic imaging shows restoration of distal RCA flow. (c) Postintervention angiogram view showing successful PCI of the RCA with deployment of a new drug‐eluting stent.

**Figure:**
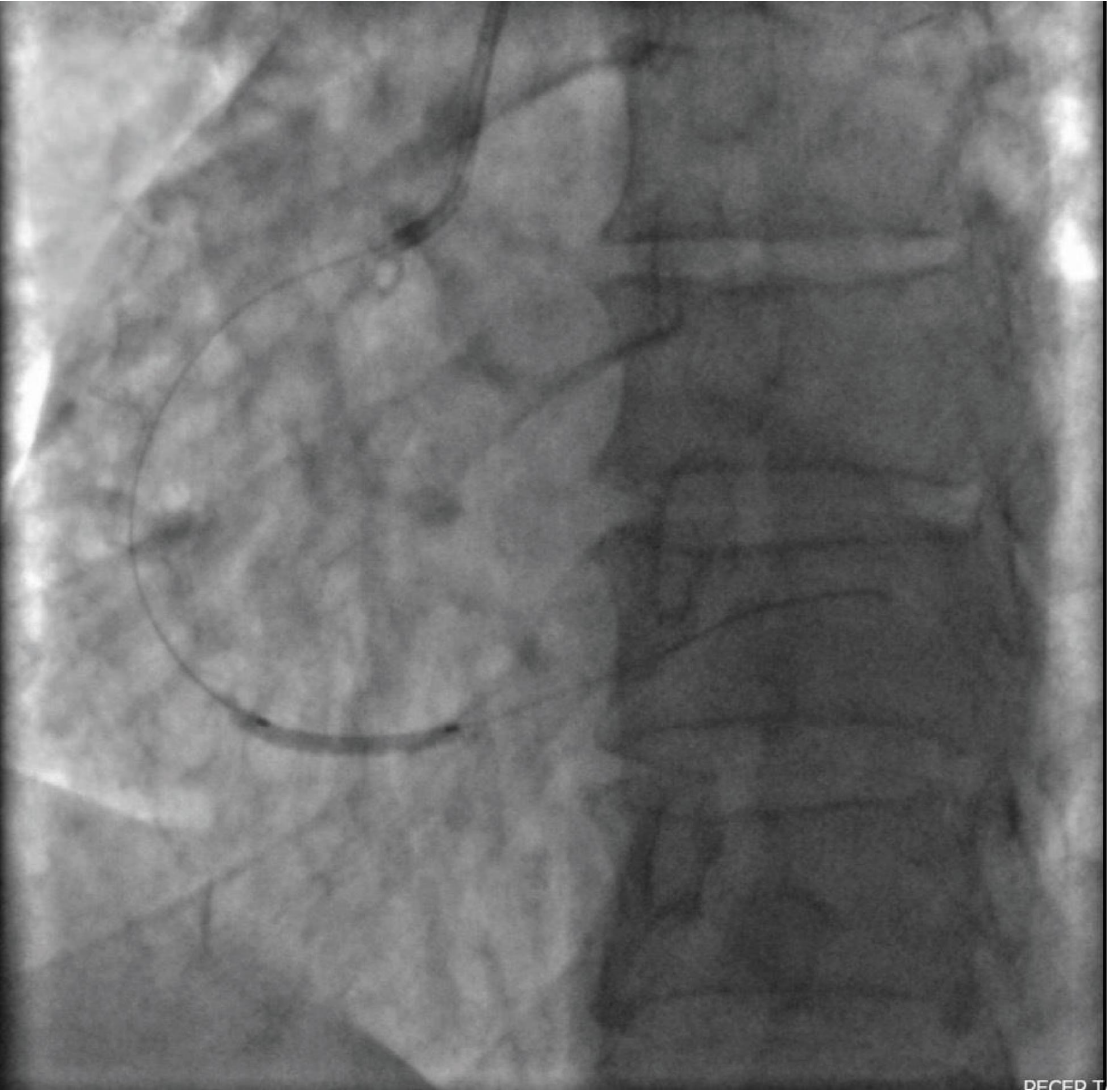
(a)

**Figure:**
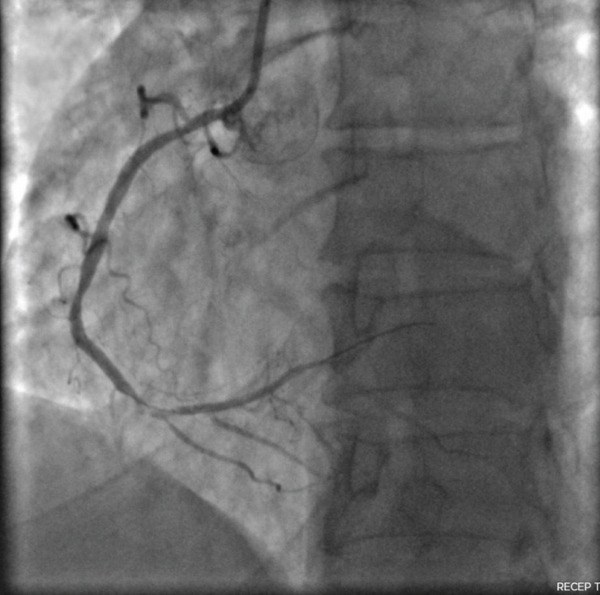
(b)

**Figure:**
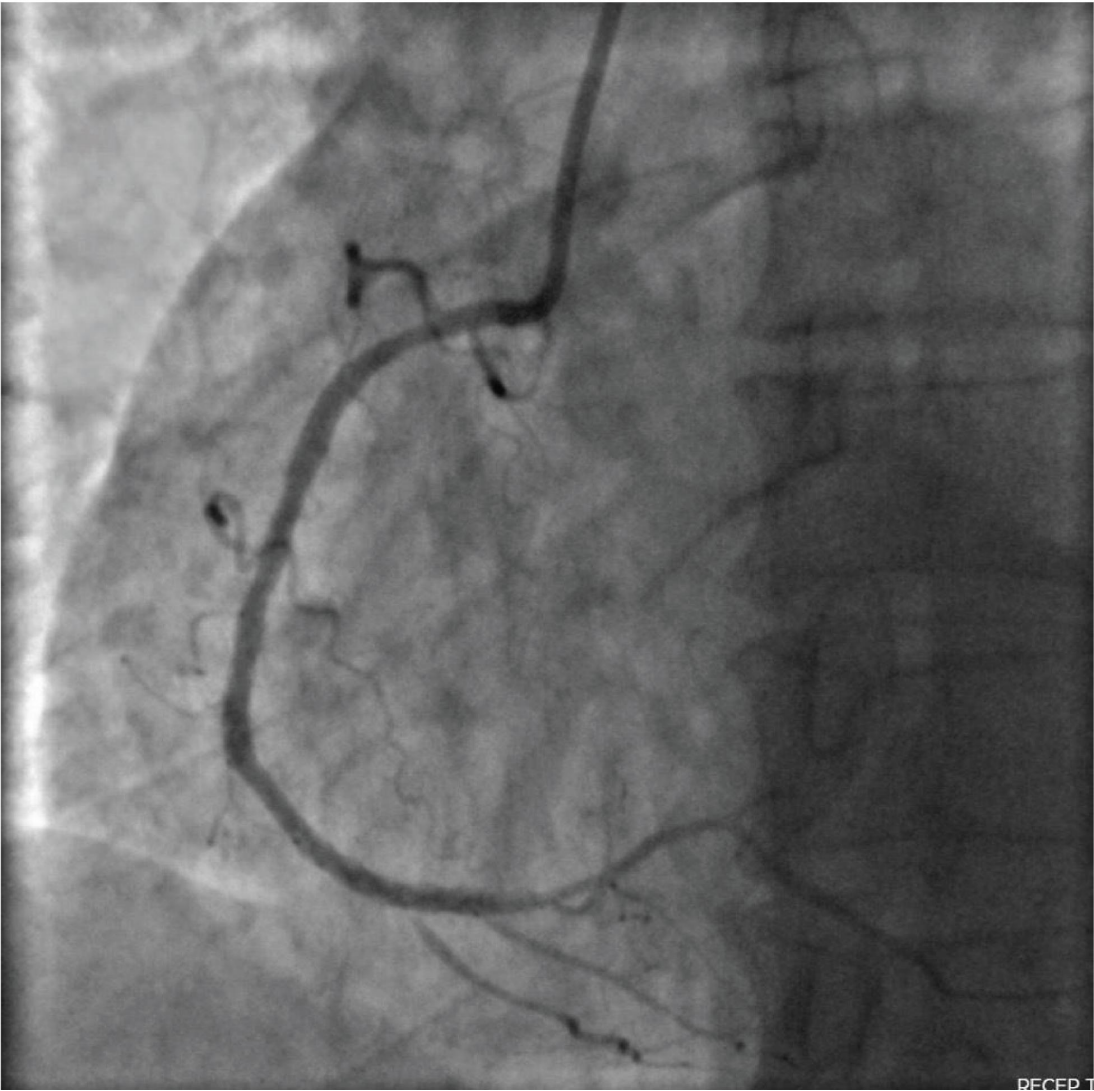
(c)

**Figure 3:**
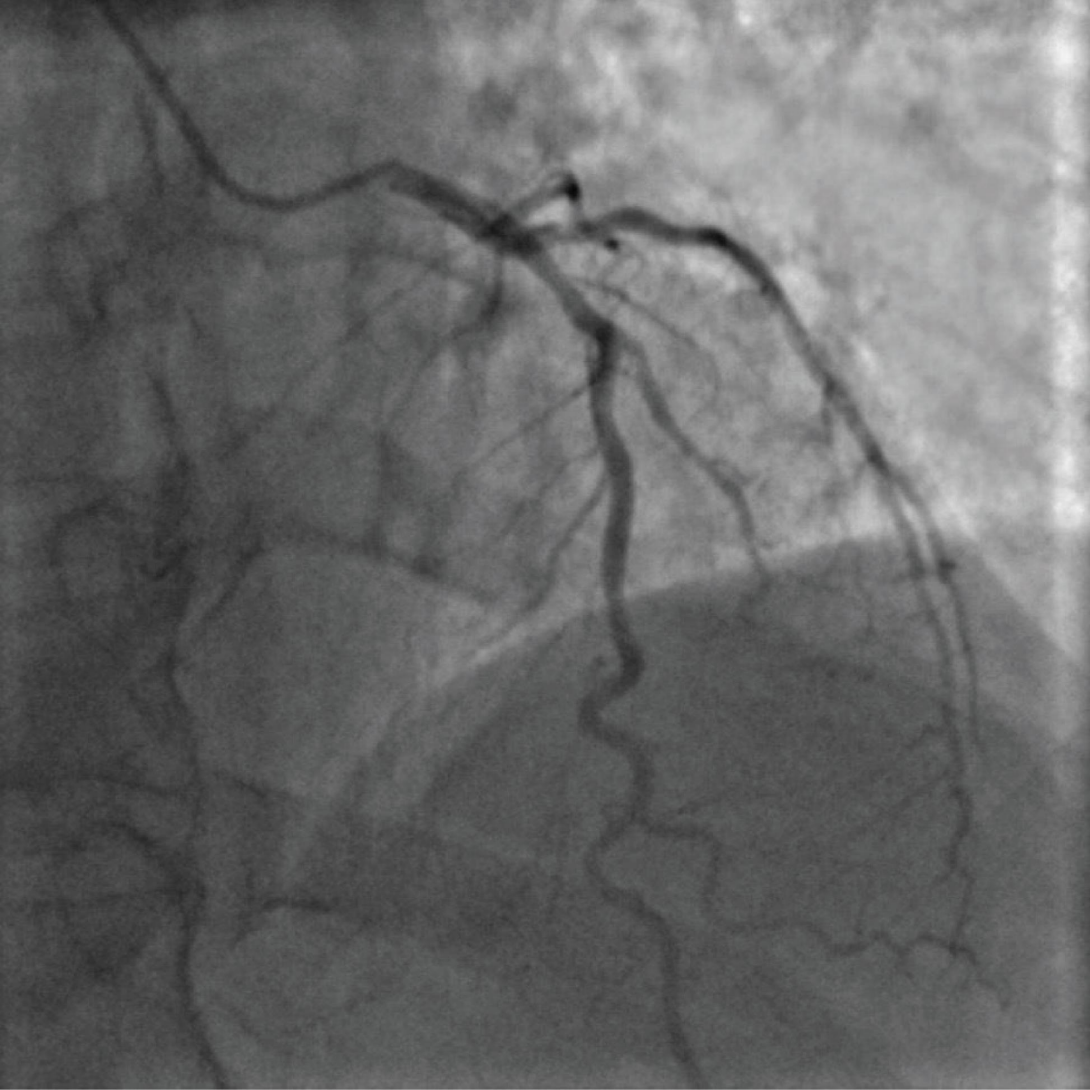
The right anterior oblique (RAO) cranial angiographic view shows the left anterior descending (LAD) artery and the circumflex (Cx) artery with no significant lesions.

Balloon angioplasty was performed in the distal RCA (Figure 2a), resulting in restoration of blood flow (Figure 2b). A drug‐eluting stent was subsequently deployed in the distal RCA (Figure 2c), whereas the left anterior descending (LAD) and circumflex (Cx) arteries showed no significant lesions (Figure 3). The patient′s clinical course was uneventful, and he was discharged 1 day later on dual antiplatelet therapy, planned for a duration of 12 months.

## 3. Discussion

MI classically presents with chest pain, dyspnea, diaphoresis, and nausea. However, atypical presentations, including silent or nonchest pain symptoms, are not uncommon, particularly in elderly, diabetic, or female patients. Persistent hiccups as the *sole* presenting symptom of ST‐elevation myocardial infarction (STEMI) remain exceedingly rare, with only a few cases documented in the literature [8].

In this case, a 60‐year‐old male presented with persistent hiccups lasting 1 month, without any other classical symptoms of MI. Diagnostic evaluation revealed *Q* waves in the inferior leads on ECG, elevated troponin levels, and inferior wall hypokinesia on echocardiography. Coronary angiography confirmed total occlusion of the distal RCA, and successful percutaneous coronary intervention (PCI) with drug‐eluting stent implantation resolved the underlying ischemia [9].

The underlying mechanism for hiccups in MI is thought to involve irritation of the vagus or phrenic nerves, both of which are in close proximity to the inferior wall of the heart and diaphragm. Ischemia or inflammation in this region, particularly involving the RCA, may stimulate these nerves, triggering the hiccup reflex arc [10].

Several prior reports support this hypothesis. In a case by Nagpal et al., a 72‐year‐old man presented with intractable hiccups and was later diagnosed with an inferior STEMI due to RCA occlusion [11]. Similarly, Kumar et al. reported a case of a 55‐year‐old male whose persistent hiccups led to the diagnosis of RCA‐related STEMI, with complete resolution of hiccups following PCI [10]. Omar et al. described a diabetic patient who presented with hiccups and was found to have a silent inferior wall MI [12].

Our case aligns closely with these reports, further strengthening the association between inferior wall ischemia and hiccups as a presenting symptom. Notably, in nearly all of these cases, RCA involvement was identified, suggesting a pattern that clinicians should be aware of [10–12].

Persistent hiccups are usually attributed to gastrointestinal, neurologic, or idiopathic causes, and cardiac origins are often overlooked. This case underscores the importance of considering cardiac evaluation—particularly ECG and cardiac biomarkers—in patients with unexplained or refractory hiccups, especially in those with risk factors or abnormal findings on physical examination or imaging [8].

This case emphasizes that persistent hiccups can be an unusual but important clinical clue to underlying myocardial ischemia, particularly involving the inferior wall. Awareness of this rare presentation can prompt earlier diagnosis and intervention, potentially improving patient outcomes [10].

## Author Contributions

All authors participated equally in patient management, data collection, and paper preparation.

## Funding

No funding was received for this manuscript.

## Disclosure

They evaluated and sanctioned the final version.

## Ethics Statement

Informed consent was secured from the patient for all treatments and procedures.

## Consent

No written consent has been obtained from the patients as there is no patient identifiable data included in this case report.

## Conflicts of Interest

The authors declare no conflicts of interest.

## Data Availability

Data supporting the findings of this case report are not publicly available due to concerns regarding patient privacy and confidentiality. Further details may be available from the corresponding author upon reasonable request and with appropriate ethical approvals.
